# Visualising treatment effects in low-vision settings: Proven and potential endpoints for clinical trials of inherited retinal disease therapies

**DOI:** 10.1038/s41434-025-00552-7

**Published:** 2025-08-07

**Authors:** Arun J. Thirunavukarasu, Shabnam Raji, Jasmina Cehajic-Kapetanovic

**Affiliations:** 1 Nuffield Laboratory of Ophthalmology, Nuffield Department of Clinical Neurosciences, University of Oxford, Oxford, UK; 2 Oxford University Clinical Academic Graduate School, University of Oxford, Oxford, UK; 3 Oxford Eye Hospital, Oxford University Hospitals NHS Foundation Trust, Oxford, UK

**Keywords:** clinical trial, endpoints, inherited retinal disease, retinitis pigmentosa, randomised-control trial, gene therapy, vision tests, geographic atrophy

## Abstract

Inherited retinal diseases are a devasting and incurable cause of blindness which frequently affect patients at a young age, and developing effective treatments has been an important research priority in recent decades. Treatments must be validated in randomised-control trials, which involve measuring benefit according to prospectively defined endpoints. A wide variety of conventional clinical endpoints and emerging anatomical, physiological, and functional biomarkers may be selected. Different options may be better or worse at capturing clinically significant differences and identifying real differences between experimental groups. This review provides an overview of some proven and potential endpoints for randomised-control trials involving inherited retinal disease patients. Clinical endpoints and biomarkers are discussed, and the work required to validate biomarkers for use in trials is outlined. Unlike in general medicine, ophthalmological clinical endpoints may all be conceptualised as surrogates. Selecting optimal endpoints is essential to ensure that treatments are assessed fairly, such that resources are directed towards interventions that stand to truly benefit patients with inherited retinal diseases.

## Introduction

Inherited retinal diseases (IRDs) are frequently functionally devastating, and represent the most common cause of blindness in patients of working age in higher income countries.(1,2) IRD patients exhibit very high rates of sight impairment associated with anxiety, depression, and unemployment.(3) In cost-of-illness terms, IRDs are estimated to cost over US$15 billion per year in the United States and Canada, and over £500 million per year in the United Kingdom and Republic of Ireland.(4,5) Despite decades of research, almost all IRDs are currently untreatable.(6,7) Developing and trialling management strategies for IRDs is therefore an important research priority for patients and practitioners.(8)

Within IRDs, defects in around 300 nuclear and mitochondrial genes result in over 100 distinct diseases with significant variability in phenotype and prognosis.(9,10) Currently, genetic testing exhibits a sensitivity of between 45-75% for IRDs, meaning that up to three quarters of patients have identifiable genetic defects which could hypothetically be targeted by gene therapy treatments.(11–15) Currently, one gene therapy for an IRD has been made commercially available: Luxturna (voretigene neparvovec-rzyl) for *RPE65*-related Leber’s congenital amaurosis. Trials of other gene therapies have not yet led to regulatory approval, but a great deal of research is leading to development of further treatments which may undergo successful trials in the future.(16)

Following confirmation of safety and dosing strategy in Phase 1 and 2 clinical trials, novel therapeutics can progress to Phase 3 trials of clinical effectiveness. This generally involves randomisation of patients to either the new intervention or a control arm. The control arm should have access to best available care such that results have applicable implications regarding change to clinical practice. As most IRDs are incurable, best available care may only entail counselling and observation, ideally with a vehicle placebo to minimise performance bias and maintain blinding to isolate treatment efficacy.(7,17) Determining effect size requires prospective definition of trial endpoints by which an intervention’s efficacy (or lack thereof) may be determined. Generally, a primary endpoint is used as the defining metric of success or failure of an intervention, with secondary endpoints used to capture other potential benefits which may merit further investigation. Retrospective analysis of non-primary endpoints or *post hoc* analysis of primary endpoints is generally not accepted as sufficient evidence for regulatory approval, due to risk of bias where analytic plans are subject to flexibility in reporting and selection.(18) For this reason, clinical trials—with all of their associated expense and time—are high-risk endeavours where the primary endpoint must be carefully considered. Defining appropriate, fair, and practical endpoints is challenging, and depends on the disease of interest, patient characteristics, and logistical resources.(19)

In this review, genetic therapies for IRDs are discussed with a focus on how putative benefits of successful treatments may be measured. The problem of translating these expected benefits into appropriate trial endpoints and determining a minimal clinically important difference (MCID) is then approached in the specific context of IRDs which exhibit variable but frequently very severe phenotypes. Endpoints used in previous trials are described alongside their strengths and limitations before potential novel endpoints for future trials are outlined (Table 1).

Determining how to assay efficacy of IRD treatment is an essential precursor to trials of new therapies for incurable IRDs.

## Characteristics of IRDs and potential therapeutic strategies

### Detection of IRD genotypes and phenotypes

Genetic defects characteristic of IRDs underlie loss of function of photoreceptor cells in the retina, typically due to primary degeneration of the photoreceptors or secondary to degeneration of the underlying choroid and retinal pigmented epithelium (RPE). Phenotypes may be categorised on the basis of whether rods (in more common rod-cone dystrophies) or cones (in rarer cone-rod dystrophies) are primarily affected by disease first, with degeneration often later involving all photoreceptors.(20,21) Phenotypes relate to the distribution and function of affected rods and cones as they are affected by disease. In rod-cone dystrophies such as classical retinitis pigmentosa, patients typically present with worsening vision in dark conditions and in the periphery of their visual field.(22) In contrast, cone-rod dystrophy patients tend to present with macular symptoms such as loss of visual acuity, dyschromatopsia, and photophobia.(20) Intermediate phenotypes are common, with overlapping symptoms and signs as well as highly variable rates of progression and patient ages at onset.(23)

**Figure 1:**
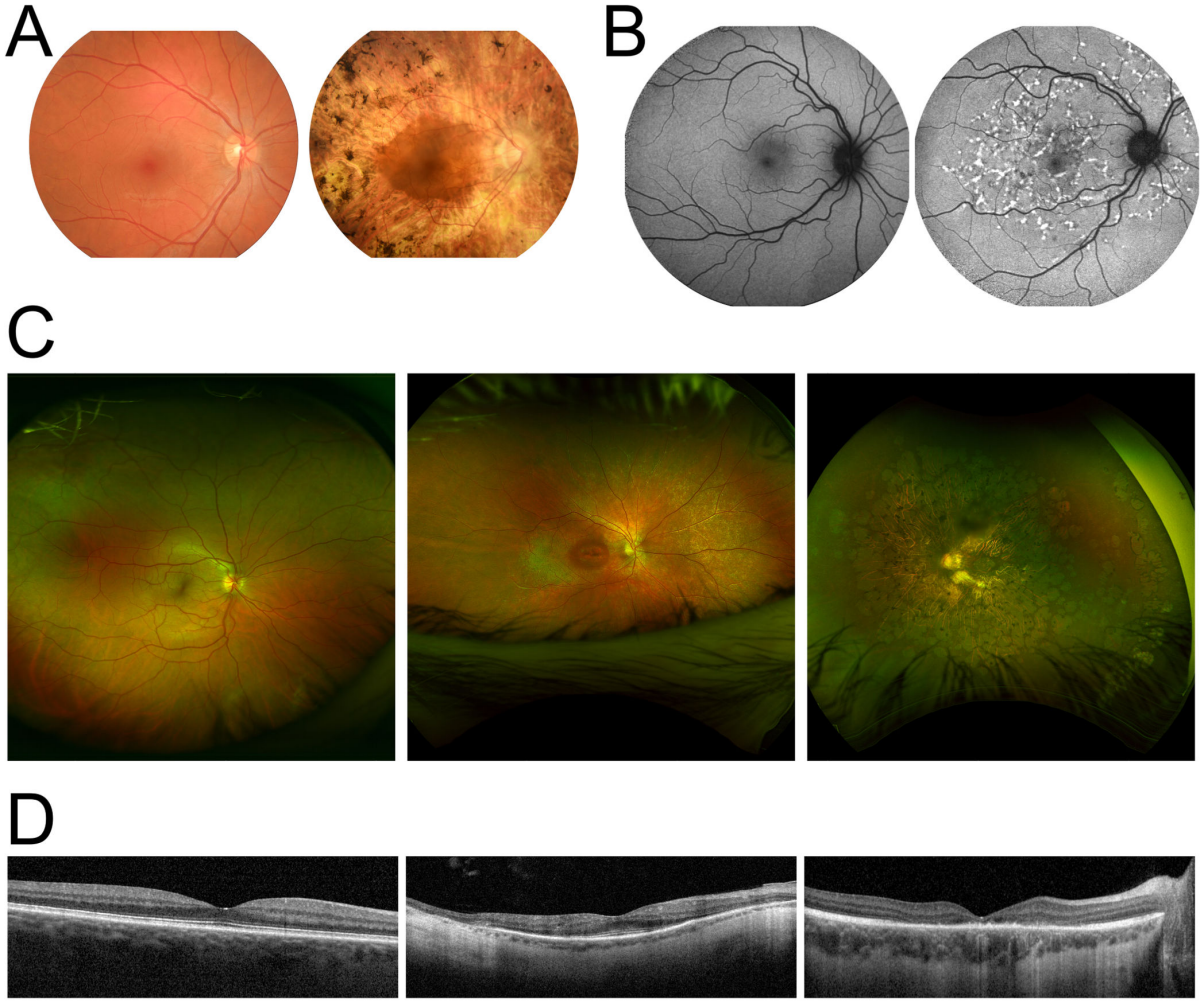
A plethora of imaging modalities may be used to characterise inherited retinal diseases. (A) Fundus photography from a healthy individual (left) depicting usual appearances of the optic disc, macula, retinal vasculature, and hue of the fundus determined by the retinal pigmented epithelium and underlying vasculature (left) and X-linked retinitis pigmentosa patient (right) with mid-peripheral pigment deposition and pale optic disc. (B) Fundus autofluorescence images in the absence of any pathology (left) and in Stargardt’s disease (right) with characteristic mottled macular flecks of hypofluorescence and hyperfluorescence, and peripapillary sparing. (C) Ultrawide field fundus photographs providing a broader view of the retina in a healthy individual (left), cone dystrophy patient (centre) with consequent macular atrophy, and RPE65-mutation associated Leber’s congenital amaurosis (right) demonstrating characteristic pigment mottling. (D) Optical coherence tomography (OCT) images of the maculae of a healthy individual (left), cone dystrophy patient (centre) with loss of the ellipsoid zone and outer photoreceptor layers apparent in and around the fovea, and X-linked retinitis pigmentosa (right) with reduced ellipsoid zone width in a more peripheral distribution.

**Figure 2:**
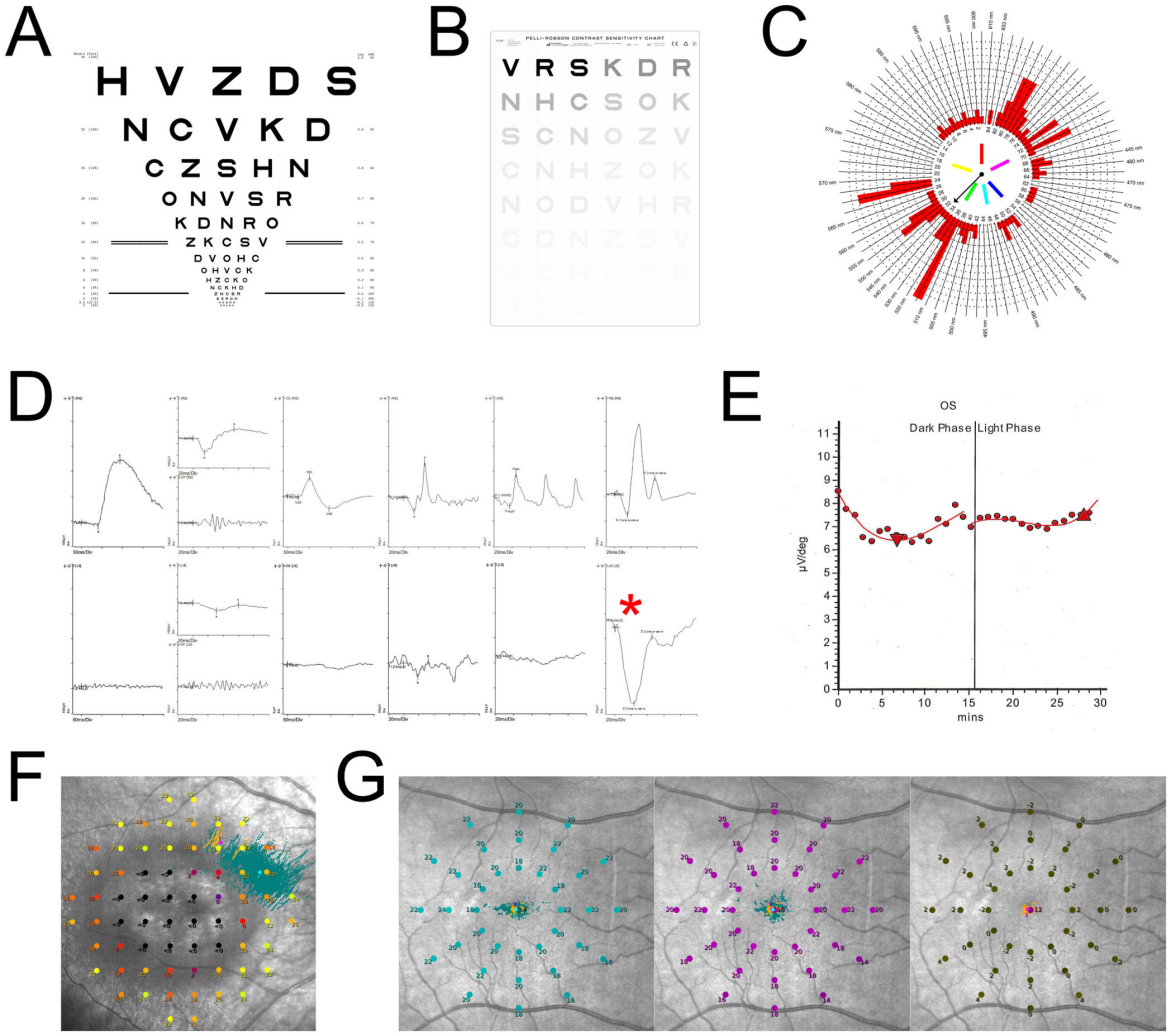
A variety of functional tests are used in the clinic and as clinical trial endpoints with IRD patients. (A) An Early Treatment of Diabetic Retinopathy Study (ETDRS) visual acuity chart read by patients at 6m; patients with macular disease cannot identify smaller letters. Patients with severe disease may be unable to identify the largest letter, with visual acuity restricted by ability to count fingers or detect light. Testing in low luminance is a means of capturing night blindless (nyctalopia) as seen in rod-dominated dystrophies. (B) The Pelli-Robson contrast sensitivity chart: many IRD patients struggle with poor contrast sensitivity, such as those affected by retinitis pigmentosa and Stargardt disease. (C) Main result plot from the Farnsworth Munsell 100-Hue Test of colour vision. Here, signs of deutan and protan defect are apparent, likely causing red-green colour-blindness. (D) Electroretinogram traces for a healthy patient (top) and N2RE mutation-driven enhanced S-cone syndrome patient. Results from seven tests are presented, from left to right: dark adapted (DA) response to 0.01 cd·s·m^-2^ flash, DA response to 3.0 cd·s·m^-2^ flash and oscillatory potentials, pattern ERG, light adapted response to 3.0 cd·s·m^-2^flash, and S-cone response. As with many IRDs, attenuated responses are seen, but the pronounced S-cone a-wave (red asterisk) is pathognomonic for enhanced S-cone syndrome. (E) Electro-oculogram trace from a patient with vitelliform macular dystrophy (Best disease), with characteristic absence of a light-induced rise. The Arden ratio may be calculated as the ratio between the light peak and the dark trough, and is reduced significantly in Best disease; often before symptoms or other clinical signs develop. (F) Mesopic microperimetry results from a patient with cone dystrophy, depicting the structure-function correlation between features of disease on fundus photography and ability to detect flashes directed at the same points. (G) Scotopic microperimetry results from a healthy control patient, showing how the difference between sensitivity to cyan (left) and red (centre) flashes are used to isolate rod function.

A wide variety of clinical tests and investigations are useful in characterising IRD phenotypes; both through direct imaging (Figure 1) and with functional tests (Figure 2).(24,25) Characteristic changes on fundoscopy or fundus photography (Figure 1A and 1C) include pigmentation, widespread atrophy, attenuated retinal vasculature, and optic disc pallor; but signs are often absent or subtle in the early stages of disease.(23) On fundus autofluorescence images (Figure 1B), changes are enhanced by lipofuscin accumulation by RPE cells due to loss of photoreceptors or RPE atrophy in progressive IRD.(26,27) Optical coherence tomography (OCT) provides useful visualisation of the inner and outer segments of photoreceptors, with thinning and disruption of the external limiting membrane (between inner and outer segments) correlating with clinical severity (Figure 1D).(27)

Full-field electroretinography (ERG) results provide quantified estimates of rod and conefunction, but capture global photoreceptor function and may therefore not be sensitive to the effects of early disease where only small portions of the retina are affected.(23,28) A wide variety of test schemata may be employed to isolate the function of different components of the neural retina (Figure 2D).(29,30) These schema vary background light intensity, flash intensity and frequency, as well as pre-adaptation steps in scotopic, mesopic, or photopic conditions.(30) Specifically, pattern ERG uses an alternating chequered light stimulus to interrogate the macular cones and ganglion cells; while multifocal ERG involves pseudorandom luminance reversal of hexagonal stimuli across the retina to localise functional deficits.(30) Alternatively, electro-oculography (EOG; Figure 2E) is effective for characterising RPE function, commonly abnormal in many IRDs although primarily used to exclude Best disease (vitelliform macular dystrophy) and other bestrophinopathies.(28,31) Functional tests such as visual acuity (best measured with the ETDRS chart shown in Figure 2A), perimetry, and contrast sensitivity (Figure 2B) are commonly used in the clinic and provide useful data to define phenotypes and progression. However, these tests frequently require adaptations for use in IRD patients with significant visual impairments or disease affecting only a limited portion of the retina. For instance, microperimetry (Figure 2F, Figure 2G) is useful to capture macular deficits where the peripheral retina is severely affected or unaffected by disease; and low luminance visual acuity has proven more sensitive in the earlier stages of IRDs.(32,33) Moreover, functional tests can be ineffective measures of progression if selected inappropriately, such as visual acuity in rod-cone dystrophies sparing the macula.(23) In addition, tracking whether treatments save or improve conventional measures of visual function frequently requires prohibitively long follow-up due to the rate of progression of disease. An understanding of IRD pathogenesis and natural history is therefore essential for accurate and informative tracking of disease progression and assessment of treatment effects. This can also help define an optimal window of opportunity for an intervention and guide inclusion criteria for trials.

Clinical signs and investigation results alone are a poor indicator of patient genotype due to the wide variety of genes implicated in IRDs, as well as the overlap exhibited between affected genes and cone- and rod-dominated dystrophies.(23,34) Many thousands of genetic variants within a few hundred genes have been identified as causes of IRD.(23) While genotype-phenotype correlation has been characterised for particular implicated genes, significant variation within individuals exhibiting similar genetic variants indicates that IRDs can be multifactorial in their pathophysiology.(23,35,36) Despite these challenges, genetic testing is the mainstay of diagnosis of IRDs. Identified variants are classified based on the likelihood of their being benign or pathogenic, using findings from previous patients, prediction tools, and functional tests if available to inform inference.(37)

### Therapeutic approaches in IRDs

IRDs remain incurable, and disease progression often leads to patients being affected by complete blindness.(6,7) Developing therapeutic strategies to slow-down or halt progression is a significant research priority, and gene therapy has emerged as a promising avenue of research.(16) Thus far, the most common genetic therapeutic strategy has been gene augmentation, with functional copies of a gene (cDNA) added via a vehicle (usually an adeno-associated virus or lentivirus) to replace a defective gene in the recipient. Gene editing is an alternative strategy where the patient’s genome is directly altered to mitigate a pathogenic variant. These approaches have been made possible by the development of the Clustered Regularly Interspace Short Palindromic Repeats (CRISPR) and CRISPR-associated (Cas) protein 9 system (CRISPR/Cas9), which has facilitated specific guidance of DNA breakage and repair-mediated editing in multiple animal models of IRDs.(38) RNA therapies have also demonstrated promise in diseases such as Leber’s congenital amaurosis.(39) The most common approach is to use antisense oligonucleotide therapies which bind to RNA sequences and thereby promote degradation, modification, or alternative splicing to reduce, modify, or regenerate proteins of interest.(40) Potential applications are diverse, ranging from editing variants to resolve photoreceptor function to targeted disruption of pathogenic DNA sequences and ablation and replacement of pathogenic variants.(38,41)

A more ambitious aim is to reverse disease progression and even cure patients by restoring vision closer to peers without an IRD. Cell therapy—providing stem cells to replace entire photoreceptors rather than defective genes—has demonstrated transient benefits to visual acuity in IRD patients, but further work is required to sustain functional improvement.(42,43) In optogenetic approaches, similar vehicles as used for gene augmentation may be used to instead transmit DNA encoding proteins such as light-sensitive native or non-native opsins to surviving photoreceptors or downstream neurons such as bipolar cells to restore sensitivity to light in advanced disease.(44–46) Alternatively, retinal or cortical implants may offer a means of bypassing the pathology of IRDs entirely, by replacing dysfunctional photoreceptors with electrical devices. While cortical implants are yet to demonstrate benefit to blind patients, retinal implants have been tested in clinical studies with modest benefits recorded in around a third of patients.(47) Further research is therefore required to improve the functionality of these systems, surgical techniques for required procedures, and thereby the overall clinical benefits of interventions for more patients.(48,49)

Previous trials of IRD therapies provide insight into the expected benefits, challenges, and results of future studies. Here, the merits and limitations of endpoints used in IRD trials are focused upon. In successful trials, halting progression or restoration of visual function may be observed, as with voretigene neparvovec-rzyl in Leber’s congenital amaurosis.(50) Patients may be unlikely to regain normal vision, but appreciable and measurable benefit is a reasonable expectation.

## Clinical endpoints and biomarkers derived from IRD phenotypes

### Clinical endpoints

In ophthalmology, clinical endpoints are objective measures of visual function, and are the gold standard for assaying benefit of a new treatment.(51,52) However, unlike endpoints used commonly in other specialties such as death and hospitalisation, an element of subjectivity affects assessment of vision. ‘Blindness’ or ‘sight impairment’ is defined variably by localities and despite legal guidance, certification as such is often a subjective clinical decision.(53) Other fields have successfully standardised definitions of clinical endpoints to maximise reliability and improve the utility of comparisons between separate trials.(54) Using available tests of vision, similar initiatives could improve the validity of clinical trial results by establishing uniform benchmarks of clinical benefit rather than relying on manufacturers and investigators with competing interests to engineer bespoke endpoints based on interventions’ likely strengths and limitations. However, any framework must account for the diverse effects on vision of ophthalmological pathology, with specific difficulties arising in IRDs due to phenotypic variation between and even within specific genotypes, as well as relatively small cohorts of patients exhibiting similar mutations and previous exposure to experimental treatments.

The most common clinical endpoints in ophthalmology are derived from visual acuity testing or perimetry (visual field testing), which provide results amenable to semi-automated processing and conventional statistical analysis.(19,55,56) However, these can be unsuitable for IRD trials due to the spectrum of associated phenotypes. Visual acuity is frequently too poor for conventional assessment with the ETDRS chart (Figure 2A), and perimetry tends not to change where sensitivity has been completely lost.(50,57) Moreover, in low acuity populations, floor effects can impede capturing of further deterioration or improvement.(58–60) Conversely, IRDs with less effect on central vision are unlikely to manifest with measurable changes during the course of a trial. However, best-corrected visual acuity (BCVA) is a primary endpoint in various completed and ongoing gene therapy trials for IRDs.(16) In the largest ocular gene therapy trial to date, of timrepigene emparvovec for choroideremia, the primary endpoint of a ≥15-letter ETDRS improvement was not met, despite many more treated than untreated patients exhibiting a ≥10-letter improvement.(61) Although these endpoints are reasonably reliable, inter-operator variability (such as the degree to which patients are pushed to read as far as possible) and patients’ cooperativeness lead to measurement noise which reduces statistical power.(62,63) Time required to conduct vision assessment rigorously, resources such as well-trained clinicians, and increased sample sizes required to identify benefit consequently impede design and conduct of clinical trials. Where therapies target a specific mutation or gene, small numbers of patients introduce a significant challenge to accrue sufficient statistical power to capture any benefit provided by the intervention. Many alternative functional biomarkers have been developed and these may graduate to acceptance as clinical endpoints with further validation work.

The Multi-Luminance Mobility Test (MLMT) has been used in rod-cone dystrophy trials. In the MLMT, videos of participants navigating an obstacle course at multiple light levels are graded by trained evaluators to determine visual performance.(50,64) Notably, the MLMT was the primary endpoint in the pivotal trial of Luxturna, the only current ocular gene therapy with regulatory approval.(50) More holistic but subjective endpoints such as MLMT or other vision-dependent tasks with measurable performance may be the most effective way to gauge visual performance of IRD trial participants, but robust blinding is critical to avoid observer bias.(65) However, the MLMT and other similarly designed obstacle courses are rarely used in real-world clinical practice due to its labour intensive protocol and requirement for physical space, raising questions about whether and how treatment effects can be monitored outside clinical trial settings.

Alternative clinical endpoints include patient-reported outcome measures (PROMs) to assay patients’ visual experience in daily life through survey questions.(66–68) Despite availability of PROMs being specifically designed for IRD patients, these have only ever been used as secondary endpoints in IRD trials, likely due to perceived subjectivity and difficulties mitigating measurement noise.(16,66–68) PROM tools are highly variable, implementation is often methodologically flawed, and quality of life itself lacks a universal definition.(69) Moreover, the PROMs frequently exhibit low reliability and variation in scores based on the method of administration.(70) PROMs also depend on patient compliance and thorough follow-up, increasing the administrative burden of a clinical trial.(71) Further development may focus on designing instruments to collect more information, and adopting impartial appraisal of patient responses. For instance, participants could provide free text responses to questions about their visual lives rather than ticking boxes or giving ratings in response to a limited number of statements. Labour intensive analysis of responses could be facilitated by machine learning approaches to automate appraisal and perhaps increase reliability and reproducibility.(72)

### Biomarkers and surrogate endpoints

Validated biomarkers may be used as surrogate endpoints if they predict clinical outcomes, or as novel clinical endpoints.(19,73) They are used for their convenience, particularly where conventional endpoints are expensive, impractical, or slow-changing. Commonly used examples in ophthalmology include intraocular pressure (IOP), which has been used as a surrogate endpoint in glaucoma to overcome issues of slow progression in visual changes despite IOP frequently not correlating with pathology.(74,75) Here, biomarkers and surrogate endpoints are categorised as either anatomical, physiological, or functional.

Surrogate outcome measures have only rarely been used in IRD trials as primary endpoints, but examples include SeaSTAR, a trial of Emixustat for Stargardt’s Disease.(76) SeaSTAR employed an anatomical surrogate endpoint—area of macular atrophy—as a biomarker of disease severity based on previous validation studies showing that fundus autofluorescence measurements of the area macular atrophy correlate with phenotype and progression (Figure 1B).(77) Similar endpoints have been used in pivotal trials of therapies targeting geographic atrophy in age-related macular degeneration.(78,79) There are many other potential anatomical endpoints for IRD trials derived from fundus photography (Figure 1A) and OCT (Figure 1D), ranging from photoreceptor inner/outer segment ratio to change in the ellipsoid zone area. By mitigating lens aberration, adaptive optics offer a means of increasing the resolution of ocular imaging to the level of individual photoreceptors, which may produce a rich source of novel biomarkers with greater clinical utility.(80) Adaptive optical techniques have been studied in a variety of IRD settings, most commonly in achromatopsia, choroideremia, Usher syndrome, and Stargardt disease; but measurements have never been used as a primary endpoint in a clinical trial.(16,80)

Electrodiagnostic tests offer another means of measuring visual function by capturing physiological responses to light stimuli (Figure 2D). The b-wave recovery rate on electroretinography has been used as a primary endpoint in Stargardt disease trials, as it depends on RPE-65 function.(81) Full-field electroretinography features are sensitive to disease progression in many rod-cone dystrophies before patients report symptoms, and also correlate with visual acuity degradation.(29,82) This could be of great utility for endpoint design in trials evaluating treatments directed towards patients with early-stage disease, who may have the greatest prospect of preserving good vision. There may be similar prospects for using electrophysiological tests in cone dystrophy trials as some features are pathognomonic of particular syndromes (Figure 2D), but disease progression may be captured with less sensitivity.(82) Other physiological biomarkers stem from the developing model of disease pathophysiology. These range from vitreoretinal leukocytes to pro-inflammatory cytokines, and associations with disease progression are well documented.(83) However, these markers exhibit poor correlation with clinical assessments and patients’ visual experience, so are unlikely to be used as trial endpoints to demonstrate clinical benefits of a new therapy.(83)

Finally, functional tests offer a potential means of capturing benefits to patients’ visual lives. Some—such as MLMT and perimetry mean deviation—are already accepted as clinical endpoints. Many options are derived from established clinical tests. Low luminance visual acuity most commonly involves a standard ETDRS chart (Figure 2A) with neutral density filters, and appears to be a useful predictor of progression and indicator of disease severity in many IRDs.(32) Proposed adaptations to existing mobility tasks such as MLMT include adoption of virtual reality versions, which may entail fewer demands for personnel and space.(84,85) Alternatively, full-field stimulus threshold (FST) testing challenges patients to identify pulses of light of variable intensities, and has become a widely used test to monitor post-Luxturna treatment disease progression in Leber’s congenital amaurosis, as the primary endpoint of its corresponding trial, MLMT, has proven too impractical for routine clinical use.(86–88) FST has already featured as a secondary endpoint in clinical trials of Luxturna, and may be considered as a primary endpoint in future trials, due to its practicality and clinical utility.(50,89)

Microperimetry is an adaptation of longer established visual field testing where the sensitivity of the macular region of the retina is specifically tested. Many microperimetry-derived endpoints are beginning to be used in IRD clinical trials.(33) A variation of the test performed in scotopic luminance conditions enables isolation of dark-adapted photoreceptor (*i.e*. predominantly rod) sensitivities within the macula (Figure 2G), which is highly pertinent to severe IRDs where nyctalopia is the presenting and predominant symptom.(90) Scotopic microperimetry has been used to assess the efficacy of gene therapy in terms of rod function, although it is yet to be used in randomised control trials as a formal endpoint.(91) Conversely, mesopic microperimetry captures cone function (Figure 2F), and early applications in the context of age-related macular degeneration may translate to cone-dominated dystrophies.(92)

## Translating biomarkers into validated clinical and surrogate endpoints

Biomarkers cannot always be relied upon as accurate indicators of visual function (and therefore of treatment efficacy), because patients lose vision in a wide variety of patterns with inconsistent effects on reported quality of life.(93,94) However, many of the biomarkers described above are comparable to accepted clinical outcomes: contrived (but usually non-invasive) clinical measurements with limited relation to the day-to-day visual lives of patients. It may be argued that all clinical endpoints in ophthalmology are functional surrogates for the ‘true’ variable of interest: holistic visual function in daily life. Crude measures such as visual acuity and mean sensitivity on perimetry do not replicate visual challenges patient experience, despite strong and voluminous data suggesting that results correlate with visual life quality.(19) More elaborate endpoints such as MLMT performance are still limited by finite possibilities and challenges, even if adapted in VR, and therefore fall short of replicating holistic visual ability.

**Figure 3:**
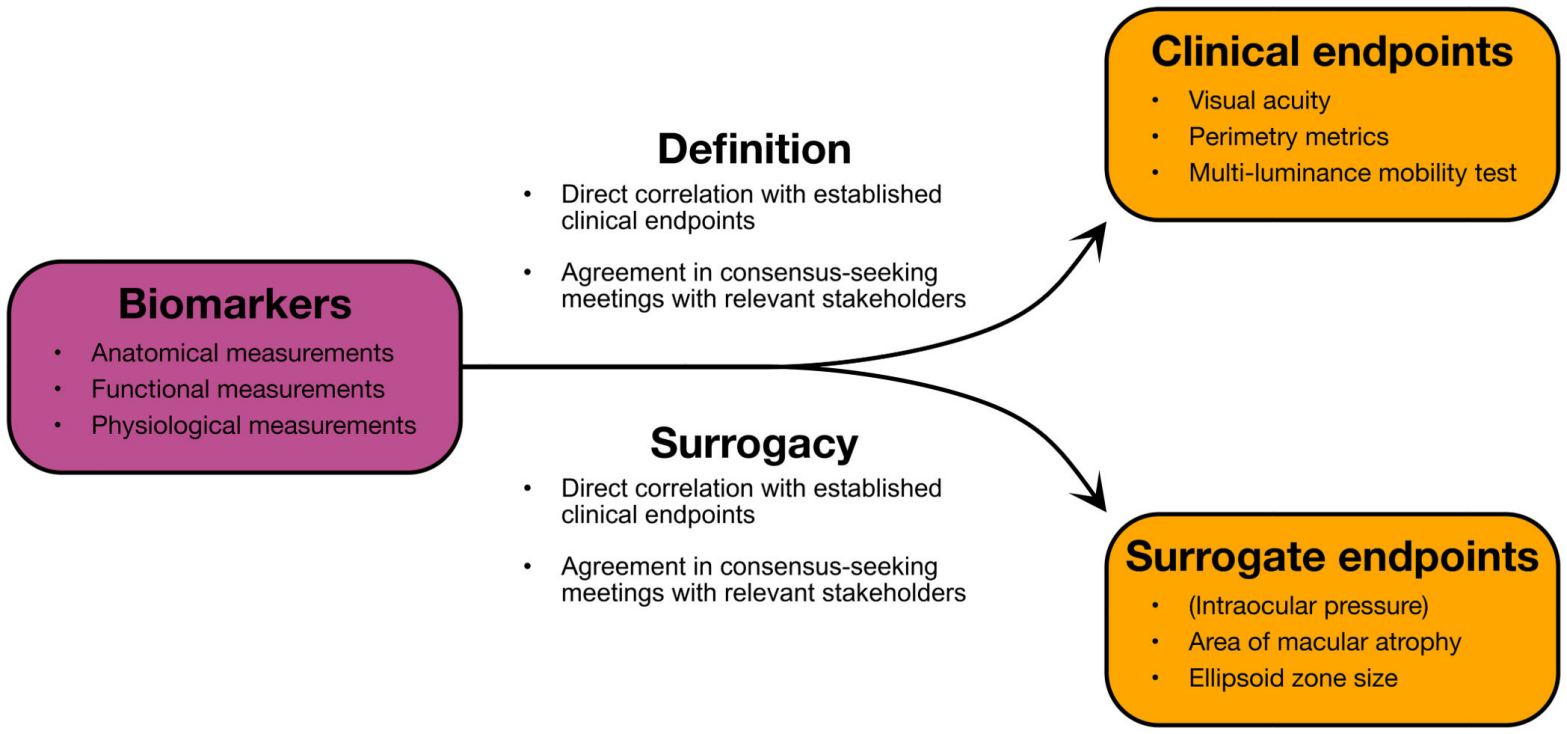
Translating biomarkers into new primary endpoints for clinical trials of IRD treatments. There are two main routes for translation: surrogacy and definition of novel clinical endpoints. Validation as a surrogate outcome requires association between the novel measurement and an established clinical endpoint such as visual acuity. This may be undertaken through clinical trials (by employing novel measurements as secondary outcomes) or purpose-designed longitudinal studies. Alternatively, definition of a novel clinical endpoint requires close correlation between the proposed measurement and established clinical outcome, or consensus meetings with all relevant stakeholders to define a new aspect of disease progression. In general surrogate outcomes may offer more convenience in trial design as they may require shorter follow-up periods or smaller sample sizes, but clinical outcomes are direct measurements of disease progression with less doubt about the clinical relevance of observed differences. Intraocular pressure is bracketed as it does not pertain to IRDs but is a archetypal example of a surrogate endpoint in wide use.

Endpoints derived from visual acuity, perimetry, contrast sensitivity, and colour vision assessment have become accepted by regulators in part due to their long established use in clinical practice.(95) However, the unique challenges presented by IRDs have been recognised with novel endpoints such as MLMT and area of geographic atrophy establishing themselves as primary endpoints for clinical trials of novel therapeutics.(50,77) Other biomarkers require validation data to convince regulators that derived endpoints correspond directly to IRD severity or predict progression as defined by other, already accepted, endpoints. There are two main validation routes for justifying novel primary endpoints in trials (Figure 3).

The first route is surrogacy. Surrogate endpoints correlate well enough with established clinical endpoints to replace them as primary trial endpoints, and are often used due to superior convenience. The classical example of a surrogate endpoint in ophthalmology is intra-ocular pressure (IOP) used in lieu of visual acuity or perimetry-based endpoints in glaucoma trials.(75) As many novel endpoints feature as secondary endpoints in IRD trials, outcomes that predict deterioration in established clinical outcomes should be explored as potential surrogates.(16) In addition, purpose-designed longitudinal studies to observe the natural history of IRDs while regularly taking a variety of measurements can similarly allow predictive variables to be identified and further validated as surrogate endpoints.(96) In the NIGHT study, a plethora of potential trial endpoints were monitored in choroideremia patients across time.(97) Retinal sensitivity, central ellipsoid zone area (on OCT), and total area of fundus autofluorescence were more sensitive markers of disease progression than best corrected visual acuity and therefore have potential as surrogate endpoints.(97) A systematic review of similar studies in X-linked retinitis pigmentosa patients concluded that ellipsoid zone width (on OCT) and outer ring area (on fundus autofluorescence) were the most robust biomarkers with potential for use as trial endpoints.(98) However, these outcomes exhibited limited ability to track progression in a 24 month observational study of X-linked retinitis pigmentosa patients, indicating that other biomarkers may demonstrate more potential to disrupt trial endpoints in this population.(99)

The second route to validation is definition as a novel clinical endpoint. As many novel endpoints have featured as secondary endpoints in IRD trials (as well as in trials of other ophthalmological conditions), it is possible to determine which of these endpoints offer a useful way of assaying benefit in terms of existing clinical endpoints by measuring their direct correlation.(16) Novel clinical endpoints may also be validated in purpose-designed studies, as has been undertaken for MLMT. MLMT performance correlates with visual impairment in terms of visual acuity and perimetry results, justifying its use as a primary endpoint.(64) Novel endpoints may also capture different aspects of disease progression than conventional endpoints—such as holistic quality of life in PROMs—and consensus-seeking meetings with clinicians, drug developers, researchers, and regulators is necessary to determine whether these are appropriate for IRDs. Decisions should be specific in their scope (*i.e*. which diseases and stages of progression) and based on measurement reliability, association with quality of life, and biological plausibility.(95) Similar initiatives have been instrumental for innovating clinical trial design in age-related macular degeneration, diabetic retinopathy, glaucoma, and other diseases.(95,100,101)

There is some overlap in the routes to validation of new primary trial endpoints, but surrogate endpoints are generally predictive of clinical outcomes (often in the future), while clinical endpoints are direct in-the-moment measurements of disease severity or progression.(19) Surrogate endpoints may therefore offer logistical advantages in clinical trial design, such as shorter follow-up without the requirement to wait for measurable disease progression, or reduced sample size as significant differences are observed sooner. These advantages are particularly important in IRD trials as eligible patients may be scarce, with associated limitations in terms of sample size, follow-up time, and measurement noise. Clinical endpoints are direct measurements of disease progression and are therefore generally preferable, as they offer less doubt about the clinical significance of observed differences between experimental groups.(19,74) However, detectable differences do not necessarily correspond to meaningful changes in vision from the patient perspective, and further work is necessary to define minimal clinically important differences.(102)

## Conclusion: Specific work is required to develop, validate, and select appropriate endpoints

Defining endpoints in clinical trials of IRD treatments is a significant biological, practical, and statistical challenge. Endpoints have a remarkable influence on trial results and must balance competing priorities of sensitively capturing the MCID while reliably overcoming measurement noise where expected benefit is modest. Conventional clinical endpoints in ophthalmology often translate poorly in IRD patients, but vision tests such as best-corrected visual acuity, perimetry, and contrast sensitivity have been used to derive primary and secondary endpoints in many IRD trials.(16) Alternative clinical endpoints include MLMT and other complex visual tasks which aim to represent patients’ visual lives more faithfully.(64) A different approach would be to rely on PROMs as a more holistic measure of visual outcomes than contrived clinical assessments and measurements, but noise generated by subjectivity and variability represents a significant challenge.(71)

A plethora of anatomical, functional, and physiological biomarkers may represent useful endpoints to capture meaningful clinical effects of novel treatments for IRDs.(83) However, rigorous validation is necessary to demonstrate that these biomarkers correspond to concurrent or future visual function in terms appreciable to patients. Moreover, contemporaneous discussion with regulators is of paramount importance to ensure that new endpoints used in trial can inform decisions about whether or not treatments will be granted regulatory approval. This importance is demonstrated by the case study of pegcetacoplan for treating age-related macular degeneration, which was refused European Medicines Agency (EMA) approval despite meeting a primary endpoint of slowing geographic atrophy lesion growth in phase 3 clinical trials.(79) Discussion should consist of consensus-seeking meetings informed by observational studies which take time to generate useful results, but which may be run alongside clinical trials to maximise efficiency in a fast-moving field with emerging therapeutics and a small population of patients.

No single best endpoint exists for IRD clinical trials, due to variation in disease phenotypes, low prevalence, and practicality concerns. To select an appropriate endpoint, investigators should consider the functional consequences of disease progression and likely benefits of treatment, available statistical power and measurement noise, as well as the relationship between outcomes and appreciable visual function.

## Figures and Tables

**Table 1:** A summary of selected proven and potential endpoints for inherited retinal disease (IRD) clinical trials. Contemporaneous consultation with regulators is key to ensuring that novel endpoints are accepted by relevant stakeholders and can thereby inform decisions to grant regulatory approval. EMA = European Medicines Administration; ETDRS = Early Treatment of Diabetic Retinopathy Study; FDA = Food and Drug Administration.

Endpoint	Endpoint status	Aspect of visual function assessed	Strengths as an endpoint	Weaknesses as an endpoint	Examples of regulatory approval in the context of IRDs
Best corrected visual acuity (BCVA; using ETDRS chart)	Clinical endpoint	Central foveal acuity	Standardised, widely used, reliable, semi-automated processing, conventional statistical analysis	Inter-operator variability, poor suitability for very poor acuity or non-central vision IRDs	Historically approved primary endpoint for various common retinal disease such as diabetic retinopathy and wet AMD trials and more recently adopted in IRD gene therapy trials *e.g.* the STAR trial for choroideremia (NCT03496012)
Low luminance visual acuity (with neutral density filters)	Clinical endpoint	Central foveal acuity (with potential parafoveal input)	Predictive, practical adaptation of existing tests; potentially more sensitive in earlier stages foveal dysfunction	Not yet formalized in trials, requires further validation	Approved primary endpoint by FDA in the VISTA trial for *RPGR*-related X-linked retinitis pigmentosa (NCT04850118)
Contrast sensitivity (Pelli-Robson)	Clinical endpoint	Central contrast function	Better correlate of real-world function, potentially more sensitive in earlier stages foveal dysfunction, newer tests able to measure contrast sensitivity at a range of spatial frequencies	Inter-operator variability, less standardisation than ETDRS BCVA	Approved as secondary endpoint in the POLARIS trial for Stargardt Disease Type 1 (NCT06435000)
Perimetry (visual field testing)	Functional biomarker/Clinical endpoint	Central and peripheral vision	Established, semi-automated, amenable to conventional statistical analysis	Insensitive when sensitivity already lost, limited change detection in some IRDs	Approved secondary endpoint in Japan for *RPGR*-related X-linked retinitis pigmentosa trial (NCT05926583)
Microperimetry (mesopic and scotopic)	Clinical endpoint	Macular sensitivity	Dark/light-adapted testing, sensitive, targeted macular assessment with fundus tracking	Evolving application as a formal primary endpoint	Approved secondary endpoint (mesopic microperimetry) in some gene therapy trials e.g. XIRIUS for *RPGR*-related X-linked retinitis pigmentosa (NCT03116113). Approved primary endpoint (mesopic microperimetry) by EMA in the VISTA trial for *RPGR*-related X-linked retinitis pigmentosa (NCT04850118).
Multi-Luminance Mobility Test (MLMT)	Clinical endpoint	Low-luminance mobility	Captures real-world visual performance, holistic, accepted primary endpoint in pivotal trial	Labour-intensive, space-demanding, observer bias risk, rarely used in real-world practice, impractical for routine use	Approved primary endpoint in pivotal trial of Luxturna for Leber’s congenital amaurosis (NCT00999609)
Virtual reality mobility assessment	Functional biomarker/Emerging clinical endpoint	Low-luminance mobility	Less resource-intensive than physical mobility courses, reduces space/personnel needs	Emerging experimental test, needs development and validation	Approved as secondary efficacy endpoint only in trials for *RPGR-*related X-linked retinitis pigmentosa (NCT04794101)
Patient-reported outcome measures (PROMs)	Functional biomarker/Subjective clinical endpoint	Patient-experienced visual function, Quality of life	Captures subjective experience, direct patient input, reflects daily life	Perceived subjectivity, methodological variability, low reliability, high admin burden, compliance-dependent, analysis challenges	Approved in various IRD trials as secondary endpoints only
Full-field Stimulus Threshold (FST)	Functional biomarker/Clinical endpoint	Global retinal sensitivity	Practical, widely used post-treatment, adaptable as primary endpoint	Secondary role so far, requires further validation as primary outcome	Approved primary endpoint in Japan for *RPE65*-associated dystrophy trial (NCT04516369), but not by FDA or EMA. Approved secondary endpoint in trials for Leber’s congenital amaurosis post-Luxturna monitoring (NCT00999609)
Fundus autofluorescence (area of macular atrophy)	Anatomical biomarker/Surrogate endpoint	Macular structural integrity	Anatomically validated, correlates with phenotype/progression, objective measurement	Indirectly related to vision, needs validation as surrogate for functional outcomes, technical imaging demands	Recently approved primary endpoint in geographic atrophy AMD trials OAKS and DERBY (NCT03525600, NCT03525613) and since adopted for IRDs *e.g.* SeaSTAR for Stargardt’s Disease (NCT03772665)
